# Statin uses and mortality in colorectal cancer patients: An updated systematic review and meta‐analysis

**DOI:** 10.1002/cam4.2151

**Published:** 2019-05-08

**Authors:** Yue Li, Xingkang He, Yu’e Ding, Hongyang Chen, Leimin Sun

**Affiliations:** ^1^ Department of Gastroenterology Zhejiang University Medical School, Sir Run Run Shaw Hospital, School of Medicine, Zhejiang University Hangzhou Zhejiang China

**Keywords:** all‐cause mortality, cancer‐specific mortality, colorectal cancer, meta‐analysis, Statins

## Abstract

**Background:**

Colorectal cancer (CRC) remains one of the most common types of cancer and a leading cause of death worldwide. Previous studies indicated that statins may have a potential protective effect on CRC.

**Methods:**

We conducted this meta‐analysis to systematically assess the overall and cancer‐specific survival benefit of statin uses on CRC patients. Related references were identified through PubMed, the Cochrane Library, Web of Science, EMBASE, and SCOPUS from inception to August 2017. Adjusted hazard ratios (HRs) were adopted to calculate summary hazard ratios (HRs) with 95% confidence intervals (95% CIs), using a random‐effects model.

**Results:**

Total fourteen studies involving 130 994 patients were included in this meta‐analysis. Six studies reported the association between pre‐diagnosis statin uses and CRC mortality, while 11 studies investigated mortality in patients using statins after CRC diagnosis. For pre‐diagnosis statin uses, the pooled HR of all‐cause mortality (ACM) was 0.85 (95% CI, 0.79‐0.92) and the pooled HR of cancer‐specific mortality (CSM) was 0.82 (95% CI, 0.79‐0.86). In terms of post‐diagnosis statin uses, the pooled HR of ACM was 0.86 (95% CI, 0.76‐0.98), and the pooled HR of CSM was 0.79 (95% CI, 0.70‐0.89). For post‐diagnosis statin uses, there is no difference in ACM when stratified by KRAS gene (KRAS) mutation status. Results of ACM and CSM did not markedly alter in other subgroup analyses.

**Conclusion:**

Our meta‐analysis demonstrates that both pre‐diagnosis and post‐diagnosis statin uses are associated with reduced ACM and CSM for CRC patients.

## 1 INTRODUCTION

Colorectal cancer (CRC) ranked third in terms of incidence and second in terms of mortality. It is estimated to have over 1.8 million new cases and cause 881 000 deaths in 2018.^1^ Many colorectal carcinomas develop through an adenoma‐carcinoma sequence for years,^2^ which makes clinical intervention and prognosis improvements possible and meanwhile essential. Nonsteroid anti‐inflammatory drugs, especially aspirin, have been shown to reduce both the incidence and the mortality of CRC in several studies.^3^, ^4^ However, taking the possible bleeding complications of aspirin and the clinical burden of CRC into account, other attractive chemopreventive agents are warranted.

Statins, the inhibitors of 3‐hydroxy‐3‐methylglutaryl coenzyme‐A (HMG‐CoA) reductase, are cholesterol‐lowering agents most commonly prescribed worldwide. Besides their lipid‐lowering effects, various researches have revealed their unexpected preventive effects on tumor development and progression via HMG‐CoA reductase‐independent pathway and HMG‐CoA reductase‐dependent pathway.^5^, ^6^ Underlying mechanisms, including suppression of tumor growth, induction of apoptosis, and inhibition of angiogenesis were engaged.^6^

While many preclinical researches on cells and animals have indicated the positive effects of statins on CRC, such as increasing intracellular oxidative stress, inducing apoptosis and augmenting chemosensitivity,^7^, ^8^ whether statin uses are positively correlated with the survival of CRC patients in clinic is controversial. No consensus concerning the prognostic effects of statins on CRC has been reached so far.^11^, ^12^ Therefore, we conducted this meta‐analysis to assess the overall and cancer‐specific survival benefits of statin uses on CRC patients. Our results may provide further insights into clinical applications of statins on CRC patients.

## 2 METHODS

This systematic review and meta‐analysis were performed in accordance with Preferred Reporting Items for Systematic Reviews and Meta‐analyses.^13^ It was registered with the PROSPERO international prospective register of systematic reviews (http://www.crd.york.ac.uk/PROSPERO, CRD42017074280).

### 2.1 Search strategy

We identified related references through searches of PubMed, the Cochrane Library, Web of Science, EMBASE, and SCOPUS from inception to August 2017, using search strategies (Table S1). All references of related articles and relevant reviews were screened manually to further identify potentially relevant studies.

### 2.2 Study selection and data extraction

The following criteria for eligibility among studies were set before selection of references: (a) the exposure of interest was statin uses before or after diagnosis of CRC, (b) case‐control or cohort studies, (c) outcomes of interest were all‐cause death or cancer‐specific death, (d) articles were published in English, (e) when several articles were published by the same authors or group, the newest or most informative article was selected. Exclusion criteria were the following: (a) no information on all‐cause or cancer‐specific survival, (b) letters to editor or commentary, reviews, (c) clinical studies reporting odds ratios (ORs) or risk ratios, or only univariate analyses. Two investigators extracted following data from the eligible articles independently: the name of first author, year of publication, origin of the study, follow‐up period, patient number, study design, patient characteristics, statin uses, risk estimates and corresponding 95% confidence intervals (95% CIs), and covariates adjusted for in the multivariable analysis. Discrepancies were resolved by consensus, involving a third investigator.

### 2.3 Quality assessment and statistical method

The methodological quality of included observational studies was independently determined by the Newcastle‐Ottawa scale (NOS),^14^ which was based on three aspects of selection, comparability, and exposure/outcome. Two investigators independently completed the quality assessments and considered studies with a score of 7 or greater as high quality. Any disagreements were discussed with a third reviewer. We adopted adjusted hazard ratios (HRs) to calculate summary hazard ratios (HRs) with 95% CIs, using a random‐effects model. We defined post‐diagnostic statin uses as any use of statins after cancer diagnosis. Statistical heterogeneity across studies was estimated by Cochrane *X* ^2^ and *I* ^2^ statistics. A *P* value of <0.1 or *I* ^2^ >50% were considered as substantial heterogeneity across studies. In addition, we conducted predetermined subgroup analyses based on tumor site, tumor stage, baseline therapy, and KRAS mutation status. A two‐tailed *P* value <0.05 was considered significantly. All statistical analyses were analyzed by Stata (version 11.0; StataCorp, College Station, TX).

## 3 RESULTS

### 3.1 Characteristics and quality assessment of included study

**Figure 1:**
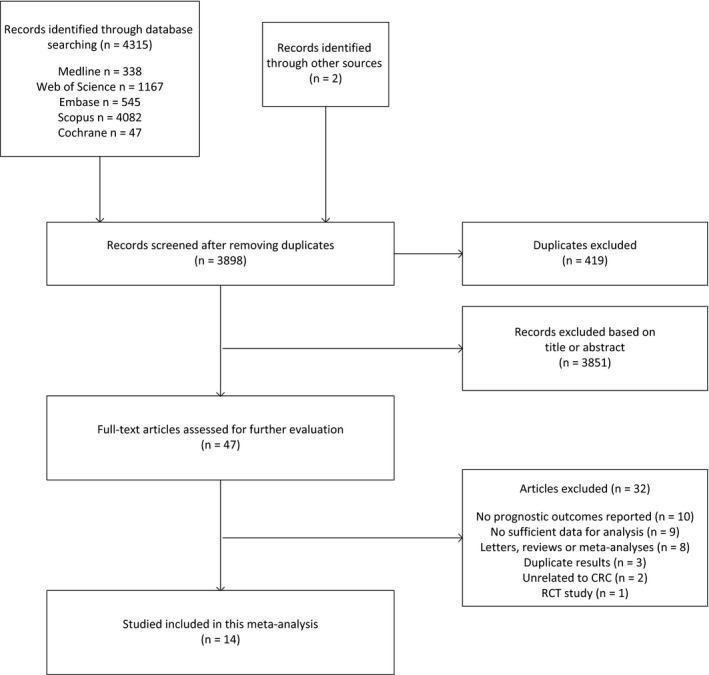
Detailed flow diagram of study selection process

The detailed flow chart of study selection process was summarized in Figure 1. Totally, 4317 potentially relevant references were identified from initial search strategy. Four hundred and nineteen articles were excluded after duplication and remaining 3898 were screened based on abstracts and titles. After exclusion of unrelated articles, 47 full‐text articles were further reviewed according to preset inclusion and exclusion criteria. Finally, 14 studies involving 130 994 patients met our criteria and were included in this meta‐analysis.^11^, ^12^, ^15^, ^16^ Detailed characteristics of the included studies were summarized in Table 1. Generally, six studies involving 86 622 patients reported survival outcomes for patients of pre‐diagnostic statin uses,^11^, ^15^, ^17^, ^18^, ^22^, ^23^, ^25^ while 11 studies involving 44 322 patients investigated survival outcomes related to post‐diagnostic statin uses.^11^, ^12^, ^16^, ^17^, ^19^, ^20^, ^21^, ^23^ Eight studies were conducted in Europe (United Kingdom, Denmark, Netherland, Germany),^11^, ^12^, ^17^, ^18^, ^20^, ^23^, ^24^, ^26^ four in North America,^15^, ^16^, ^19^, ^25^ and two in Asia (China, Korea).^21^, ^22^ Six studies investigated patients with stages I‐IV,^15^, ^17^, ^18^, ^19^ four with stages I‐III,^11^, ^22^, ^23^, ^25^ two with stage III,^16^, ^21^ one with stages II‐III,^24^ one with stage IV cancer.^12^ One study only included patients with rectum cancer,^19^ four studies only included patients with colon cancer,^16^, ^18^, ^24^, ^26^ while the remaining nine studies included patients with both colon cancer and rectum cancer.^11^, ^12^, ^15^, ^17^, ^20^, ^21^, ^22^ The methodological quality of all included case‐control and cohort studies was summarized (Table S2). The NOS results revealed that 13 of the 14 included studies had a score ≥7 except one study scored 6.^15^

**Table 1:** Baseline characteristics of included studies investigating survival outcomes of colorectal cancer patients using statin

Year 1st author Country	Study design	Tumor site	Statin Uses & No. of Patients	Tumor Stage	Treatment	Endpoints	Adjusted variables
2009 Sidiqui US	Retrospective	Colon Rectum	Pre: 1309	I‐IV	S+C/R	CSM	BMI, NSAIDs
2011 Ng US	Prospective	Colon	Post: 842	III	S+C	ACM DFM RFM	Age, sex, family history of CRC, baseline performance status, depth of invasion through bowel wall, number of positive lymph nodes, perineural invasion, Extravascular invasion, postoperative carcinoembryonic antigen, treatment arm, BMI, physical activity, Western pattern diet, and consistent aspirin use
2012 Lakha UK	Prospective	Colon Rectum	Pre: 277 Post: 282	I‐IV	S+CR	ACM CSM	Age, sex, region of residence, family history of cancer, past medical history of cancer, past medical history of bowel disease, BMI, smoking, physical activity, regular NSAID intake
2012 Nielsen Denmark	Prospective	Colon	Pre: 43487	I‐IV	C/R	CSM	Age at diagnosis, cancer staging, chemotherapy, radiotherapy, cardiovascular disease before cancer, diabetes mellitus before cancer, birth year, sex, descent, highest obtained level of education, size of residential area
2013 Mace US	Retrospective	Rectum	Post: 407	I‐IV	S+C/R	ACM CSM DFM RFM	Age, BMI, ASA class III/IV (relative to I/II), and pathological stage
2014 Cardwell UK	Prospective	Colon Rectum	Pre: 14026 Post: 7657	I‐III I‐III	S+C/R S+C/R	ACM CSM	Year of diagnosis, age at diagnosis, sex, stage, surgery within 6 mo, radiotherapy within 6 mo, chemotherapy within 6 mo, site, deprivation, comorbidities before diagnosis, and other medication use after diagnosis as time‐varying covariates
2014 Krens Netherland	Retrospective	Colon Rectum	Post: 529	IV	C	ACM PFM	Age, prior adjuvant therapy, aspirin use, >1 organ affected by metastatic spread, treatment arm, KRAS mutation status, and a KRAS*statin interaction term
2015 Hoffmeister Germany	Prospective	Colon Rectum	Post: 2697	I‐IV	S+C/R	ACM CSM RFM	Age at diagnosis, sex, Union Internationale Contre le Cancer stage, location of CRC, surgery, neoadjuvant treatment, chemotherapy, radiotherapy, BMI, lifetime pack‐years of active smoking, average lifetime physical activity, ever regular use of NSAIDs, ever use of HRT(women), previous large bowel endoscopy, diabetes, hyperlipidemia, myocardial infarction, stroke, heart failure, participation in general health check‐ups, and for a time‐dependent effect of chemotherapy (chemotherapy*log[time])
2015 Kim Korea	Retrospective	Colon Rectum	Post: 686	III	S+C/R	CSM DFM	Age; sex; comorbidity; pre‐diagnosis aspirin use; medication; cancer site; initial stage; pathological differentiation
2015 Shao China	Prospective	Colon Rectum	Pre: 17115	I‐III	S+C/R	ACM CSM	Age; sex; diagnosis year; physician visits and hospitalization 1 y before diagnosis; exposure to aspirin, other NSAIDs, insulin, oral antidiabetic drugs, angiotensin‐converting enzyme inhibitors, and angiotensin II receptor blockers; and the aforementioned comorbidities
2016 Gray UK	Prospective	Colon Rectum	Pre: 10408 Post: 8391	I‐III	S+C/R	ACM CSM	Age, sex, year of diagnosis, deprivation, site, comorbidities and aspirin use, stage, grade, and cancer treatment within 6 mo
2017 Gray UK	Retrospective	Colon	Post: 680	II‐III	S+C	ACM CSM	Age, gender, year of diagnosis, grade, MSI status, ECOG performance status, family history of CRC, adjuvant chemotherapy use, stage, and aspirin use, Charlson Comorbidity Index score
2017 Lash US	NA	Colon Rectum	Post: 21152	I‐III	S+C/R	ACM CSM RFM	Age at colorectal cancer diagnosis, sex, calendar period of diagnosis, AJCC stage at diagnosis, surgical urgency, receipt of neoadjuvant or adjuvant chemotherapy, receipt of radiation therapy, Charlson comorbidity score (31) at diagnosis (0, 1 or 2, or ≥3), and history of inflammatory bowel disease at diagnosis
2017 Voorneveld Netherlands	Retrospective	Colon	Post: 999	I‐IV	S+C	ACM CSM	Sex, age, comorbidity, year of incidence, histological grade, stage, microsatellite status, chemotherapy, and aspirin use

ACM = All‐cause mortality; AJCC = American Joint Committee on Cancer; BMI = Body mass index; C = Chemotherapy; CRC = Colorectal cancer; CSM = Cancer‐specific mortality; DFM = Disease‐free mortality; HRT = hormone replacement therapy; NA = Not available; NSAIDs = Nonsteroid anti‐inflammatory drugs; Post = Post‐diagnosis; Pre = Pre‐diagnosis; R = Radiotherapy; RCT = Randomized controlled trial; RFM = Recurrence‐free mortality; S = Surgery.

### 3.2 Association between pre‐diagnostic statin uses and mortality

Six studies reported the association between pre‐diagnosis statin use and mortality in CRC patients. One study^15^ was excluded as it only reported OR of cancer‐specific mortality (CSM). The remaining five studies with 85 313 patients were then analyzed.

### 3.3 Pre‐diagnostic statin uses and all‐cause mortality

**Figure 2:**
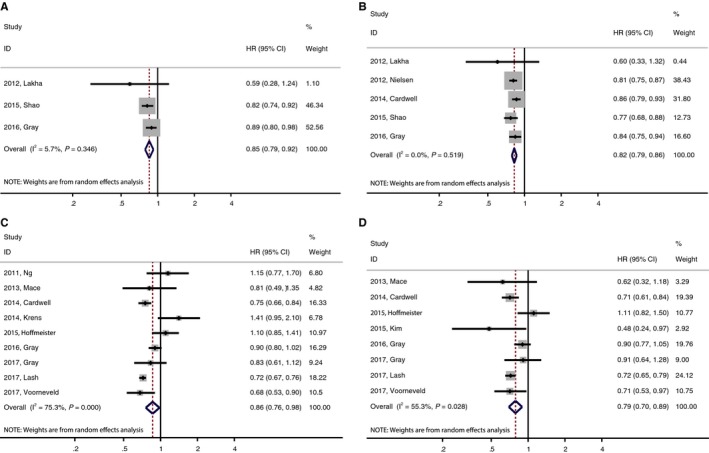
Meta‐analysis of the association between statin uses and mortality of colorectal cancer (CRC) patients. (A) Pre‐diagnosis statin uses and all‐cause mortality. (B) Pre‐diagnosis statin uses and cancer‐specific mortality. (C) Post‐diagnosis statin uses and all‐cause mortality. (D) Post‐diagnosis statin uses and cancer‐specific mortality

For all‐cause mortality (ACM), the pooled HR was 0.85 (95% CI, 0.79‐0.92) 0.80 (95% CI, 0.70‐0.91) with a minor heterogeneity (*I* ^2^ = 5.7%, *P* = 0.346), indicating that pre‐diagnosis statin uses significantly lowered the risk of ACM (Figure 2A). In the subgroup analysis stratified by country, tumor site, tumor stage and therapy, both the heterogeneity and the result of ACM did not markedly alter (data not shown). No evidence of publication bias was observed in any analyses using Begg's (*P* = 1.000) and Egger's tests (*P* = 0.494) (data not shown).

### 3.4 Pre‐diagnostic statin uses and CSM

In terms of CSM, the pooled HR was 0.82 (95% CI, 0.79‐0.86) with no heterogeneity (*I* ^2^ = 0.0%, *P* = 0.519), suggesting that pre‐diagnosis statin uses were associated with a 18% lower CSM (Figure 2B). In the subgroup analysis stratified by country, tumor site, tumor stage and therapy, both the heterogeneity and the result of CSM did not markedly alter (data not shown). No evidence of publication bias was observed in any analyses using Begg's (*P* = 0.462) and Egger's tests (*P* = 0.293) (data not shown).

### 3.5 Association between post‐diagnostic statin uses and mortality

Eleven studies investigated the association between post‐diagnosis statin uses and mortality in CRC patients. One study^17^ was excluded due to its risk of immortal time bias. The remaining 10 studies with 44 040 patients were further analyzed.

### 3.6 Post‐diagnostic statin uses and ACM

**Figure 3:**
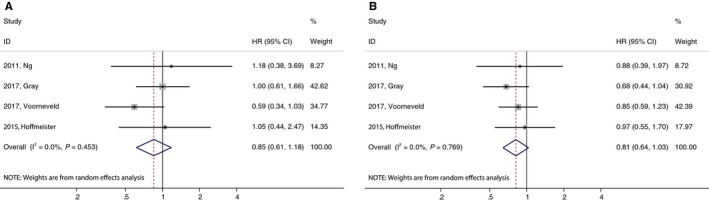
Subgroup analysis of the association between post‐diagnosis statin uses and mortality of colorectal cancer (CRC) patients. (A) All‐cause mortality among KRAS‐mutated CRC patients. (B) All‐cause mortality among KRAS wild‐type CRC patients

For ACM, the pooled HR was 0.86 (95% CI, 0.76‐0.98) with a considerable heterogeneity (*I* ^2^ = 75.3%, *P* = 0.000) (Figure 2C). In the subgroup analysis stratified by country, tumor site, tumor stage and therapy, the result of ACM did not markedly alter (data not shown). Subgroup analysis by KRAS mutation status revealed no statistical differences in ACM between statin users and nonusers. For KRAS‐mutated CRC patients, the pooled HR of ACM was 0.85 (95% CI 0.61‐1.18) (Figure 3A); for KRAS wild‐type CRC patients, the pooled HR of ACM was 0.81 (95% CI 0.64‐1.03). When stratified by tumor type, the heterogeneity decreased (*I* ^2^ = 57.5%, *P* = 0.095) in patients with colon cancer. In other subgroups, the heterogeneity did not significantly change. No evidence of publication bias was observed in any analyses using Begg's (*P* = 0.251) and Egger's tests (*P* = 0.053) (data not shown).

### 3.7 Post‐diagnostic statin uses and CSM

In terms of CSM, the pooled HR of eight studies was 0.79 (95% CI, 0.70‐0.89) with a significant heterogeneity (*I* ^2^ = 55.3%, *P* = 0.028), suggesting that statin uses reduced the risk of CSM (Figure 2D). In the subgroup analysis stratified by country, tumor site, tumor stage and therapy, the result of CSM did not markedly alter (data not shown). When stratified by tumor type, the heterogeneity decreased (*I* ^2^ = 10.6%, *P* = 0.290) in patients with colon cancer. In other subgroups, the heterogeneity did not significantly change. No evidence of publication bias was observed in any analyses using Begg's (*P* = 0.902) and Egger's tests (*P* = 0.794) (data not shown).

### 3.8 Sensitivity analysis

Omitting single study in sensitivity analysis did not markedly alter the overall results of ACM and CSM with pre‐ and post‐diagnosis statin uses (data not shown). However, when calculating CSM with post‐diagnosis statin uses, the heterogeneity decreased (*I* ^2^ = 37.8%, *P* = 0.141) after omitting 2015, Hoffmeister. In other subgroups, the heterogeneity did not significantly change (data not shown).

## 4 DISCUSSION

The present meta‐analysis demonstrates that statin uses both before and after CRC diagnosis improved the overall and cancer‐specific survival for CRC patients. These effects persisted even after subgroup analysis stratified by country, tumor site, tumor stage and therapy. Our results may be important for making further clinical decisions for CRC patients.

Several studies in vitro and vivo have strongly indicated the anticancer effects of statins on CRC. Role of statins as an adjuvant agent for CRC treatment has been suggested in many preclinical animal models. Experiment on colon‐26 cell lines model in vivo by Feleszko et al reveals that, combined treatment with lovastatin and doxorubicin resulted in significant retardation of tumor growth, as compared with either of the agents alone.^27^ In the MIN mice model, it is indicated that atorvastatin was effective in significantly slowing the growth of colon cancer cell xenografts due to increased levels of apoptosis.^28^ Cho et al shows that, in the colitis‐associated colon cancer model, simvastatin inhibited colon cancer development by the induction of apoptosis and the suppression of angiogenesis.^29^ There are various underlying molecular mechanisms of statins on CRC. Statins are competitive inhibitors of HMG‐CoA reductase, the rate‐limiting enzyme of mevalonate pathway. The mevalonate pathway produces various end products, including farnesyl pyrophosphate (FPP) and geranyl pyrophosphate (GPP), which are involved in cellular proliferation, angiogenesis, and anti‐apoptosis via inducing isoprenylation of the intracellular G‐proteins.^30^ Reduced levels of mevalonate via statins, therefore, results in antiproliferative, proapoptotic, anti‐angiogenic, and anti‐invasive effects.^30^ Furthermore, HMG‐CoA reductase‐independent mechanisms are also indicated. Statins inhibit DNA methyltransferase activity, demethylate bone morphogenetic protein 2 promoters and activate the bone morphogenetic protein pathway, which increases apoptosis and promotes differentiation in CRC cells.^31^

Besides, several recent researches have suggested the possible synergistic effects of statins and EGFR inhibitors on CRC with KRAS mutations.^32^, ^33^ KRAS mutations are estimated to account for approximately 30%‐40% of CRC patients nonresponsive for monoclonal antibodies targeting EGFR.^34^ As an essential element of the EGFR signaling pathway, KRAS can acquire activating mutations in exon 2, thus rendering EGFR inhibitors ineffective.^35^ The activation of KRAS protein could initiate several downstream signaling cascades, including Raf/MAPK, Rac/Rho, PI3K/PKB, thus promoting cell proliferation, migration and survival.^36^ By inhibiting the production of FPP and geranylgeranyl pyrophosphate (GGP) which are essential for Ras prenylation, statins could interfere with Ras functional localization and thus inhibiting the downstream signaling pathways.^36^ Therefore, in this study, the prognostic effects of statins were analyzed based on KRAS mutation status. Due to limited number, only four studies were included. No improvement on overall survival was observed when stratified by KRAS mutation status for post‐diagnosis statin uses. Further studies need to explore the association between KRAS status and survival benefit of statin uses. However, our study does show a possible trend toward a reduction in ACM among KRAS mutant CRC patients using statins after diagnosis.

The beneficial effects of statins on CRC prognosis could be attributed to both its cancer‐prevention effects and its potential role on cancer adjuvant therapy. Pre‐diagnosis statin uses have been demonstrated to be preventive for colorectal adenoma and CRC. In a retrospective cohort study following 3587 patients with histologically confirmed adenomatous polyps (APs), statin uses were associated with less polyp number, smaller polyp size, lower incidence rate of advanced APs.^37^ A recently conducted meta‐analysis involving 13 239 patients showed that, statins did not significantly affect the risk of any adenoma but was inversely correlated with the risk of advanced colorectal adenoma.^38^ Moreover, in a meta‐analysis evaluating clinical CRC risk, the pooled results from 42 researches showed a modest reduction of CRC incidence after statin uses.^39^ The role of statins on cancer adjuvant therapy has also been studied in clinical. A retrospective, case‐control study from US reported a less advanced tumor stage, a lower frequency of distant metastases, and a higher survival rate for male CRC patients taking statins.^15^ Also, three well‐designed retrospective cohort studies all reported improved pathological complete response to neoadjuvant chemoradiation in rectal cancer patients with statin uses.^19^, ^40^, ^41^

To our knowledge, there were four meta‐analysis^23^, ^42^, ^43^ so far that analyzed the association between statin uses and prognosis of CRC. Compared with the previous analyses, our meta‐analysis updated some important information, including three new studies involving 22 831 patients, which accounts for over 20% of all patients included. In three former studies,^42^, ^43^ there are several limitations interfering with the conclusion. First, data from several studies^16^, ^17^ recorded in Cai et al^42^ and Ling et al^43^ were inconsistent with original data. Second, the OR in Siddiqui et al^15^ was misused as HR for analysis, and the potential immortal time bias in Lakha et al^17^ was overlooked. Nielsen et al^18^ was calculated twice in Ling et al.^43^ Also, essential data from several studies^41^, ^45^, ^46^ included in Gray et al^23^ have not been publicly published yet, therefore, quality assessment could not be done, and the data accuracy cannot be guaranteed. Hence, these studies were excluded in our meta‐analysis.

There are some limitations in our study. Firstly, heterogeneity does not markedly alter in subgroup analysis. Therefore, other potential sources of heterogeneity, such as using of nonsteroid anti‐inflammatory drugs, statin dose and duration, location of CRC, pathological differentiation, should be further analyzed if data are available. Secondly, owing to the relatively small sample size, some subgroup analysis may contain few studies and conclusions might be less convincing. For instance, in subgroup analysis stratified by KRAS mutation status, only four studies were included. Results may be more stable and reliable with increasing number of studies that could be involved in future. Thirdly, dose‐response analysis was not conducted due to insufficient research data in this study. This might be an important confounding factor when estimating the effects of statins. Furthermore, due to the limited relevant researches, disease‐free survival and recurrence‐free survival were not included in our study. Up until now, available evidence is not sufficient to show any difference in disease‐free survival DFS and recurrence‐free survival RFS in CRC patients taking statin therapy. Whether statins own cancer‐directed benefits or not should be further discussed, and statins prescription should not be a routine for CRC prevention or treatment by now.

## 5 CONCLUSIONS

In conclusion, our meta‐analysis demonstrates that both pre‐diagnosis and post‐diagnosis statin uses are associated with reduced ACM and CSM for CRC patients. Considering that statins are low cost and wildly used agents worldwide, we believe our updated meta‐analysis can provide new insights into optimizing adjuvant treatment of CRC. Further clinical studies, especially RCTs and basic studies investigating KRAS mutations, are expected to be conducted to confirm the role of statins in CRC treatment.

## CONFLICTS OF INTERESTS

The authors declare that there is no conflict of interest regarding the publication of this paper.
