# Supplementary material for: Statin uses and mortality in colorectal cancer patients: An updated systematic review and meta‐analysis
Source: Cancer Med. 2019 May 8;8(6):3305–13. doi: 10.1002/cam4.2151 (PMC6558478; doi:10.1002/cam4.2151)
Supplement: Supplementary file 1 [file CAM4-8-3305-s001.docx]

**Search strategies used in Pubmed**

| 1 | "Hydroxymethylglutaryl-CoA Reductase Inhibitors"[Mesh] |
| --- | --- |
| 2 | Statin$ |
| 3 | HMG CoA Statin$ |
| 4 | HMG-CoA Statin$ |
| 5 | HMG CoA Reductase Inhibitor$ |
| 6 | HMG-CoA Reductase Inhibitor$ |
| 7 | Hydroxymethylglutaryl CoA Inhibitor$ |
| 8 | Hydroxymethylglutaryl-CoA Inhibitor$ |
| 9 | Hydroxymethylglutaryl Coenzyme A Inhibitor$ |
| 10 | Hydroxymethylglutaryl-Coenzyme A Inhibitor$ |
| 11 | Hydroxymethylglutaryl CoA Reductase Inhibitor$ |
| 12 | Hydroxymethylglutaryl-CoA Reductase Inhibitor$ |
| 13 | HMGCR inhibitor$ |
| 14 | or/1-13 |
| 15 | "Colonic Neoplasms"[Mesh] |
| 16 | "Colorectal Neoplasms"[Mesh] |
| 17 | "Rectal Neoplasms"[Mesh] |
| 18 | Colon Neoplasm$ |
| 19 | Colon tumor$ |
| 20 | Colon tumour$ |
| 21 | Colon carcinoma$ |
| 22 | Colonic Neoplasm$ |
| 23 | Colon Cancer$ |
| 24 | Colonic Cancer$ |
| 25 | Colorectal cancer$ |
| 26 | Colorectum cancer$ |
| 27 | Colorectal neoplasm$ |
| 28 | Colorectum neoplasm$ |
| 29 | Colorectal tumor$ |
| 30 | Colorectal tumour$ |
| 31 | Colorectal carcinoma$ |
| 32 | Cancer of the colorectum |
| 33 | Cancer of colorectum |
| 34 | Rectal cancer$ |
| 35 | Rectum cancer$ |
| 36 | Rectal neoplasm$ |
| 37 | Rectum neoplasm$ |
| 38 | Rectal tumor$ |
| 39 | Rectal tumour$ |
| 40 | Rectal carcinoma$ |
| 41 | Cancer of the rectum |
| 42 | Cancer of rectum |
| 43 | or/15-42 |
| 44 | 14 and 43 |

**Search strategies used in Cochrane**

| 1 | MeSH descriptor: [Hydroxymethylglutaryl-CoA Reductase Inhibitors] explode all trees |
| --- | --- |
| 2 | "Statin" or "Statins" or "HMG CoA Statin" or "HMG CoA Statins" or "HMG-CoA Statin" or "HMG-CoA Statins" or "HMG CoA Reductase Inhibitor" or "HMG CoA Reductase Inhibitors" or "HMG-CoA Reductase Inhibitor" or "HMG-CoA Reductase Inhibitors" or "Hydroxymethylglutaryl CoA Inhibitor" or "Hydroxymethylglutaryl CoA Inhibitors" or "Hydroxymethylglutaryl-CoA Inhibitor" or "Hydroxymethylglutaryl-CoA Inhibitors" or "Hydroxymethylglutaryl Coenzyme A Inhibitor" or "Hydroxymethylglutaryl Coenzyme A Inhibitors" or "Hydroxymethylglutaryl-Coenzyme A Inhibitor" or "Hydroxymethylglutaryl-Coenzyme A Inhibitors" or "Hydroxymethylglutaryl CoA Reductase Inhibitor" or "Hydroxymethylglutaryl CoA Reductase Inhibitors" or "Hydroxymethylglutaryl-CoA Reductase Inhibitor" or "Hydroxymethylglutaryl-CoA Reductase Inhibitors" or "HMGCR inhibitor" or "HMGCR inhibitors" |
| 3 | 1 or 2 |
| 4 | MeSH descriptor: [Colonic Neoplasms] explode all trees |
| 5 | MeSH descriptor: [Colorectal Neoplasms] explode all trees |
| 6 | MeSH descriptor: [Rectal Neoplasms] explode all trees |
| 7 | Colon Neoplasm or "Colon Neoplasms" or "Colon tumor" or "Colon tumors" or "Colon tumour" or "Colon tumours" or "Colon carcinoma" or "Colon carcinomas" or "Colonic Neoplasm" or "Colonic Neoplasms" or "Colon Cancer" or "Colon Cancers" or "Colonic Cancer" or "Colonic Cancers" or "Colorectal cancers" or "Colorectal cancer" or "Colorectum cancer" or "Colorectum cancers" or "Colorectal neoplasm" or "Colorectal neoplasms" or "Colorectum neoplasms" or "Colorectum neoplasm" or "Colorectal tumor" or "Colorectal tumors" or "Colorectal tumour" or "Colorectal tumours" or "Colorectal carcinoma" or "Colorectal carcinomas" or "Cancer of the colorectum" or "Cancer of colorectum" or "Rectal cancer" or "Rectal cancers" or "Rectum cancer" or "Rectum cancers" or "Rectal neoplasm" or "Rectal neoplasms" or "Rectum neoplasm" or "Rectum neoplasms" or "Rectal tumor" or "Rectal tumors" or "Rectal tumour" or "Rectal tumours" or "Rectal carcinoma" or "Rectal carcinomas" or "Cancer of the rectum" or "Cancer of rectum" |
| 8 | 4 or 5 or 6 or 7 |
| 9 | 3 and 8 |

**Search strategies used in Web of Science**

| 1 | TS= (Statin$) OR TS= (HMG CoA Statin$) OR TS= (HMG-CoA Statin$) OR TS= (HMG CoA Reductase Inhibitor$) OR TS= (HMG-CoA Reductase Inhibitor$) OR TS= (Hydroxymethylglutaryl CoA Inhibitor$) OR TS= (Hydroxymethylglutaryl-CoA Inhibitor$) OR TS= (Hydroxymethylglutaryl Coenzyme A Inhibitor$) OR TS= (Hydroxymethylglutaryl-Coenzyme A Inhibitor$) OR TS= (Hydroxymethylglutaryl CoA Reductase Inhibitor$) OR TS= (Hydroxymethylglutaryl-CoA Reductase Inhibitor$) OR TS= (HMGCR inhibitor$) |
| --- | --- |
| 2 | TS= (Colon Neoplasm$) OR TS= (Colon tumo?r$) OR TS= (Colon carcinoma$) OR TS= (Colonic Neoplasm$) OR TS= (Colon Cancer$) OR TS= (Colonic Cancer$) OR TS= (Colorectal cancer$) OR TS= (Colorectum cancer$) OR TS= (Colorectal neoplasm$) OR TS= (Colorectum neoplasm$) OR TS= (Colorectal tumo?r$) OR TS= (Colorectal carcinoma$) OR TS= (Cancer of the colorectum) OR TS= (Cancer of colorectum) OR TS= (Rectal cancer$) OR TS= (Rectum cancer$) OR TS= (Rectal neoplasm$) OR TS= (Rectum neoplasm$) OR TS= (Rectal tumo?r$) OR TS= (Rectal carcinoma$) OR TS= (Cancer of the rectum) OR TS= (Cancer of rectum) |
| 3 | 1 and 2 |

**Search strategies used in EMBASE**

| 1 | 'statin' OR 'statins' OR 'hmg coa statin' OR 'hmg coa statins' OR 'hmg-coa statin' OR 'hmg-coa statins' OR 'hmg coa reductase inhibitor' OR 'hmg coa reductase inhibitors' OR 'hmg-coa reductase inhibitor' OR 'hmg-coa reductase inhibitors' OR 'hydroxymethylglutaryl coa inhibitor' OR 'hydroxymethylglutaryl coa inhibitors' OR 'hydroxymethylglutaryl-coa inhibitor' OR 'hydroxymethylglutaryl-coa inhibitors' OR 'hydroxymethylglutaryl coenzyme a inhibitor' OR 'hydroxymethylglutaryl coenzyme a inhibitors' OR 'hydroxymethylglutaryl-coenzyme a inhibitor' OR 'hydroxymethylglutaryl-coenzyme a inhibitors' OR 'hydroxymethylglutaryl coa reductase inhibitor' OR 'hydroxymethylglutaryl coa reductase inhibitors' OR 'hydroxymethylglutaryl-coa reductase inhibitor' OR 'hydroxymethylglutaryl-coa reductase inhibitors' OR 'hmgcr inhibitor' OR 'hmgcr inhibitors' |
| --- | --- |
| 2 | 'colon neoplasm' OR 'colon neoplasms' OR 'colon tumor' OR 'colon tumors' OR 'colon tumour' OR 'colon tumours' OR 'colon carcinoma' OR 'colon carcinomas' OR 'colonic neoplasm' OR 'colonic neoplasms' OR 'colon cancer' OR 'colon cancers' OR 'colonic cancer' OR 'colonic cancers' OR 'colorectal cancers' OR 'colorectal cancer' OR 'colorectum cancer' OR 'colorectum cancers' OR 'colorectal neoplasm' OR 'colorectal neoplasms' OR 'colorectum neoplasms' OR 'colorectum neoplasm' OR 'colorectal tumor' OR 'colorectal tumors' OR 'colorectal tumour' OR 'colorectal tumours' OR 'colorectal carcinoma' OR 'colorectal carcinomas' OR 'cancer of the colorectum' OR 'cancer of colorectum' OR 'rectal cancer' OR 'rectal cancers' OR 'rectum cancer' OR 'rectum cancers' OR 'rectal neoplasm' OR 'rectal neoplasms' OR 'rectum neoplasm' OR 'rectum neoplasms' OR 'rectal tumor' OR 'rectal tumors' OR 'rectal tumour' OR 'rectal tumours' OR 'rectal carcinoma' OR 'rectal carcinomas' OR 'cancer of the rectum' OR 'cancer of rectum' |
| 3 | 1 and 2 |

**Search strategies used in SCOPUS**

| 1 | ALL ("Statin$") OR ALL ("HMG CoA Statin$") OR ALL ("HMG-CoA Statin$") OR ALL ("HMG CoA Reductase Inhibitor$") OR ALL ("HMG-CoA Reductase Inhibitor$") OR ALL ("Hydroxymethylglutaryl CoA Inhibitor$") OR ALL ("Hydroxymethylglutaryl-CoA Inhibitor$") OR ALL ("Hydroxymethylglutaryl Coenzyme A Inhibitor$") OR ALL ("Hydroxymethylglutaryl-Coenzyme A Inhibitor$") OR ALL ("Hydroxymethylglutaryl CoA Reductase Inhibitor$") OR ALL ("Hydroxymethylglutaryl-CoA Reductase Inhibitor$") OR ALL ("HMGCR inhibitor$") |
| --- | --- |
| 2 | ALL ("Colon Neoplasm$") OR ALL ("Colon tumo?r$") OR ALL ("Colon carcinoma$") OR ALL ("Colonic Neoplasm$") OR ALL ("Colon Cancer$") OR ALL ("Colonic Cancer$") OR ALL ("Colorectal cancer$") OR ALL ("Colorectum cancer$") OR ALL ("Colorectal neoplasm$") OR ALL ("Colorectum neoplasm$") OR ALL ("Colorectal tumo?r$") OR ALL ("Colorectal carcinoma$") OR ALL ("Cancer of the colorectum") OR ALL ("Cancer of colorectum") OR ALL ("Rectal cancer$") OR ALL ("Rectum cancer$") OR ALL ("Rectal neoplasm$") OR ALL ("Rectum neoplasm$") OR ALL ("Rectal tumo?r$") OR ALL ("Rectal carcinoma$") OR ALL ("Cancer of the rectum") OR ALL ("Cancer of rectum") |
| 3 | 1 and 2 |
