# Supplementary material for: Statin uses and mortality in colorectal cancer patients: An updated systematic review and meta‐analysis
Source: Cancer Med. 2019 May 8;8(6):3305–13. doi: 10.1002/cam4.2151 (PMC6558478; doi:10.1002/cam4.2151)
Supplement: Supplementary file 2 [file CAM4-8-3305-s002.docx]

| **Newcastle-Ottawa Scale for assessing the quality of studies in meta-analysis** | | | | | | | | | |
| --- | --- | --- | --- | --- | --- | --- | --- | --- | --- |
|  | Selection | | | | Comparability | Outcome | | | Quality score |
| Study | Representativeness of the exposed cohort | Selection of the non-exposed cohort | Ascertainment of exposure | Demonstration that outcome of interest was not present at the start of study | Comparability of cohorts on the basis of the design or analysis | Assessment of outcome | Was follow-up long enough for outcomes to occur | Adequacy of follow up of cohorts |  |
| 2009, Sidiqui | / | ★ | ★ | ★ | ★ | ★ | ★ | / | 6 |
| 2011, Ng | / | ★ | ★ | ★ | ★★ | ★ | ★ | / | 7 |
| 2012, Lakha | / | ★ | ★ | ★ | ★★ | ★ | ★ | / | 7 |
| 2012, Nielsen | / | ★ | ★ | ★ | ★★ | ★ | ★ | ★ | 8 |
| 2013, Mace | / | ★ | ★ | ★ | ★★ | ★ | ★ | / | 7 |
| 2014, Cardwell | / | ★ | ★ | ★ | ★★ | ★ | ★ | / | 7 |
| 2014, Krens | / | ★ | ★ | ★ | ★★ | ★ | ★ | / | 7 |
| 2015, Hoffmeister | / | ★ | ★ | ★ | ★★ | ★ | ★ | ★ | 8 |
| 2015, Kim | / | ★ | ★ | ★ | ★★ | ★ | ★ | ★ | 8 |
| 2015, Shao | / | ★ | ★ | ★ | ★★ | ★ | ★ | / | 7 |
| 2016, Gray | / | ★ | ★ | ★ | ★★ | ★ | ★ | / | 7 |
| 2017, Gray | / | ★ | ★ | ★ | ★★ | ★ | ★ | / | 7 |
| 2017, Lash | / | ★ | ★ | ★ | ★★ | ★ | ★ | / | 7 |
| 2017, Voorneveld | / | ★ | ★ | ★ | ★★ | ★ | ★ | / | 7 |
